# Behavioural and electrophysiological correlates of sensory attenuation in the somatosensory and auditory modality within a virtual reality setup

**DOI:** 10.1038/s41598-025-30373-y

**Published:** 2025-12-23

**Authors:** Gianluigi Giannini, Till Nierhaus, Polina Soldatova, Felix Blankenburg

**Affiliations:** 1 Neurocomputation and Neuroimaging Unit (NNU), Freie Universität Berlin, Berlin, Germany; 2 Berlin School of Mind and Brain, Humboldt Universität zu Berlin, Berlin, Germany

**Keywords:** Neuroscience, Psychology, Psychology

## Abstract

Sensory attenuation is the phenomenon that self-produced stimulations are suppressed compared to externally generated ones, both at the subjective and electrophysiological level. Despite the extensive literature on this phenomenon, it remains unclear whether electrophysiological attenuations are consistent across senses and whether they do reflect subjective attenuations of perceived intensity for self-produced sensations. Therefore, the aim of the present study is twofold: first we aimed to collect behavioural and electrophysiological measures of sensory attenuation in a controlled virtual reality setup, both in the auditory and somatosensory domain. Secondly, we correlated behavioural and electrophysiological indices of sensory attenuation to formally test whether the suppression for potentials evoked by self-generated stimulations reflects the sensory suppression revealed by behavioural measures. A total of 28 participants were included to compare the intensity of a first stimulation, which was self-generated or externally administered, to a second stimulation, which was administered at rest with varying intensity. The stimulations could be either electrical pulses at the fingertip or auditory clicks. Participants were also required to undergo a control task in which no stimulation was administered. The behavioural results indicate a reduced perceived intensity for self-produced compared to externally administered stimuli for the auditory domain. In contrast, no such difference was observed for the somatosensory domain. EEG results revealed suppression of the P2 for the auditory modality and the P200 in the somatosensory modality. Furthermore, a positive correlation between the P2 suppression and subjective intensity attenuation was found for the auditory modality. Together, our results suggest that electrophysiological suppression at mid-latency components reflect the perceived subjective attenuation of self-produced stimulation. This relationship, however, might be dependent on the sensory domain.

## Introduction

In our day-to-day interactions with the environment, our brain distinguishes what is done by ourselves and what by others^1,2^. The mechanisms responsible for this include the phenomenon of sensory attenuation, where self-generated sensations compared to externally induced ones are dampened, both at the neurophysiological^3–6^ and subjective^7–11^ level.

Sensory attenuation is commonly investigated at the behavioural level by means of a comparison task. Participants are required to either self-generate or passively experience a first – *test* – stimulation, which is then compared to a second – *comparison* – stimulation administered at rest with varying intensity. After the second stimulation, participants typically indicate which stimulation was felt stronger. This comparison task allows one to calculate the value of the perceived stimulation intensity for which the test and comparison stimulations are felt identical in 50% of trials. This value is commonly referred to as threshold or point of subjective equality (PSE). When compared with the perceptual threshold at rest, lower thresholds for self-produced sensations indicate that the second stimulation is more likely to be judged as higher; i.e., the first stimulation is felt as weaker when produced by the subject^5,7,8,12–19^. Similar attenuations for self-produced stimuli have also been demonstrated by other studies that required participants to produce congruent or incongruent outcomes, with respect to previously learned action-effects. Results that employed a similar paradigm usually report lower sensitivity for congruent action-effects, suggesting that the perception of outcomes that match motor predictions is dampened compared to outcomes that do not match the predicted action consequences^9,20^. However, the literature is not unanimous in reporting attenuation effects for self-produced sensations, with evidence ranging from null effects^21,22^ to enhancement effects for self-produced sensations^16,21,23–25^.

Concerning its electrophysiological correlates, sensory attenuation is usually investigated by means of *contingent paradigms*^26^. In those setups participants are required to: (i) perform a motor act that results in a sensory stimulation, (ii) passively attend a stimulation and (iii) perform similar motor sequences without any sensory consequences. The *motor-only* condition is commonly subtracted from the *motor-and-sensory* trial average to obtain a motor-corrected potential of self-generated stimulation that is then compared to the *sensory-only* potential perceived at rest. Electroencephalography (EEG) findings have shown an attenuation of early and middle-latency sensory-evoked components at around 100 and 200ms, either in the auditory^6,23,27–34^, visual^35–38^ or both^14^ and, despite less numerous, in the somatosensory domain^39–41^.

Despite the extensive body of literature, it remains unclear whether the physiological attenuation reported by EEG studies corresponds to perceptual differences reflected in behavioural measures, especially when considering that electrophysiological results seem to be more unanimous, with only few studies that reported contrasting evidence^23,42,43^. Behavioural evidence of intensity judgment alterations for self-reduced sensation, on the other hand, is less consistent in reporting attenuations^44^. These diverse findings challenge the commonly agreed theory that electrophysiological suppressions reflect a reduction of the perceived intensity for self-produced stimuli.

To better address this conundrum, only a handful of studies have simultaneously collected both behavioural and electrophysiological measures of attenuation within the same experimental setup^23,41,43,45,46^ and only one scrutinised their statistical correlation, reporting a positive correlation between visual P2 amplitude attenuation and behavioural attenuation measured through an intensity comparison task^14^. The study, however, failed to replicate the same findings for the auditory modality, raising important questions regarding whether electrophysiological attenuations are indeed associated to a dampening of self-generated stimuli perception and whether this relationship is univocal across sensory modalities. On this matter, it is also important to outline how sensory stimulations are administered throughout the literature for each sensory modality under scrutiny in the present study. No striking differences are observed when examining studies that investigated sensory attenuation in the auditory modality whether through electrophysiological^6,14,23,27–34^ or behavioural measures^17,18,23,47^; i.e., in both pool of studies, participants were primarily required to press a button to generate an auditory stimulation. In studies that investigated sensory attenuation via behavioural measures in the somatosensory modality, however, this phenomenon has been scrutinised via force matching tasks^7,8,19^ stroke^5,12^ or haptic judgements^20,48^; i.e., sensory tasks that poorly adapts to the temporal precision required by electrophysiological recordings. It is therefore not only necessary to reconcile behavioural and electrophysiological measures in a unique setup, but also to investigate them using a common method of stimulation.

Regarding the mechanisms that may underlie this phenomenon, it is generally agreed that, upon action execution, motor areas generate an efference copy of the motor command that is used to predict the sensory consequences of an action^5,8,49^, which are also referred to as “motor predictions”^50^. If the predicted action outputs match the effective action consequences, then the incoming sensory signal (the sensorial consequence of the action) is attenuated. This picture of sensory attenuation, however, seems too simplistic to take into account the growing body of evidence that is accumulating in recent years. For instance, recent findings indicate that different factors might modulate the attenuation driven by self-producing a stimulation, such as temporal predictability, temporal control and stimulus identity prediction^51^. Therefore, if an efference copy of a motor command underlies sensory attenuation, it appears that it does not merely encompass the sensory consequences of the action itself, but also coveys more general information about the incoming – to be produced – sensory stimulation. To better understand this relationship, a previous study published by our group^39^ advocated for the use of new technologies, such as Virtual Reality (VR) to control for a plethora of possible contributing factors in the attenuation for self-generated stimuli, such as temporal predictability and attentional requirements across self-generated and externally-administered conditions. In this way, we extended the previously scarce pool of literature on the electrophysiological correlates of sensory attenuation in the somatosensory domain through electrical pulses as stimulation. To better generalize these results, and to further characterise the phenomenon of sensory attenuation, it is important to formally compare it to other modalities that have been more extensively explored in prior research, such as the auditory modality.

The first aim of the present study, therefore, was to investigate sensory attenuation both behaviourally and electrophysiologically through an intensity comparison task in VR using a common method of stimulation, i.e., electrical pulses, and to examine their statistical correlation. Secondly, to further substantiate our previous findings, we explored sensory attenuation in two different modalities: somatosensory and auditory. By doing so, we could investigate this phenomenon in the somatosensory domain through a tactile stimulation that was viable for both electrophysiological and behavioural quantification, allowing us to further explore whether electrophysiological attenuations for self-generated stimulations indeed reflect a perceived intensity reduction. We could also further validate those findings by comparing sensory attenuation across sensory modalities and could assess whether a sensory modality dependent effect exists.

We expected to find an event-related potential (ERP) attenuation for self- compared to externally-generated stimuli in the N/P100 and P200 components for the somatosensory domain. Additionally, we hypothesized that the PSE for self-generated stimulations is significantly smaller than the PSE for externally-perceived ones. Lastly, we aimed to replicate these findings in the auditory domain.

## Methods

The experiment consisted of an intensity comparison task in VR and concurrent EEG recording. The participants performed the experiment in a 3-dimensional virtual environment, in which they could either actively reach towards or passively be touched by (*move* or *stay* conditions) a virtual ball that could give them an electrical somatosensory stimulation (*touch*), an auditory stimulation (*audio*) or control (*no-stimulation*), resulting in 6 possible conditions. One second after the administration of the first stimulation, participants received a second stimulus of the same modality always at rest. Participants then had to perform an intensity comparison task, in which they had to report whether the intensity of the second stimulation was higher or lower than the first one.

### Participants

29 healthy volunteers (20–37 years old, mean: 27.79, 13 females, all right-handed), recruited from the student body of the Freie Universität Berlin and the general public, participated for monetary compensation or an equivalent in course credit. The sample size was based on previous studies investigating sensory attenuation using a similar design^14,39,43^. Written informed consent was obtained from all subjects and/or their legal guardian(s) before participating in the experiment. The study was approved by the ethics committee at the Freie Universität Berlin (003/2021), and it was performed in accordance with the declaration of Helsinki.

### Experimental setup/apparatus

The paradigm was presented in virtual reality (VR) using an Oculus Rift CV1 headset (Meta, Menlo Park, California, USA), mounted on top of a chinrest. This setup minimized electrical and mechanical artifacts generated by wearing the headset on the EEG cap^52–54^.

The administration of electrical and auditory stimuli was controlled through a data acquisition card (National Instruments Corporation, Austin, Texas, USA). Somatosensory stimuli were delivered using a DS5 isolated bipolar constant current stimulation (Digitimer Limited, Welwyn Garden City, Hertfordshire, UK) via adhesive electrodes (GVB-geliMED GmbH, Bad Segeberg, Germany) attached to the tip of the right index finger (cathode proximal, anode distal). Auditory stimuli were given through an amplifier (AS501, Dell, Round Rock, Texas, USA) connected to a pair of headphones (HD206, Sennheiser GmbH, Wedemark-Wennebostel, Germany). Both electrical and auditory stimuli consisted of rectangular pulses of 0.2 ms duration. Lastly, participants gave responses during the experiment through a set of foot pedals.

The VR scene was built using Unity v.2020.3.26f1 (Unity Technologies, San Francisco, California, USA). The scene was identical to that used in a previous experiment^39^ with the only difference that in the present scenario, a set of virtual pedals was also rendered at the bottom of the virtual environment, within field of view of participants (see below for a description of the paradigm). When subjects pressed a pedal anytime during the experiment, also the corresponding virtual pedal changed colour, resembling the pressure exerted in the real-world. Participants were instructed to keep their left foot on the left pedal and the right foot on the right pedal and to press only when prompted to do so.

Throughout the experiment, participants could control the movement of a virtual right hand by moving along the real-world table an Oculus controller mounted on a sliding support. Hand position and rotation along the three axes were recorded throughout the whole experiment with a time resolution of 85.83 Hz (SD = 6.30 Hz).

### Calibration and setup

At the start of each experimental session, participants’ somatosensory and auditory thresholds were determined by manually changing the intensity of the stimulation until participants reported feeling 5 out of 10 stimuli (threshold somatosensory = 2.01 ± 1.32 mA, threshold auditory = 66.86 ± 5.74 dB [mean ± SD]). Then, to determine a set of stimulus intensities to be used for the experimental phase, participants underwent an intensity comparison task (almost identical to the one in the experimental phase) in which they received two subsequent stimuli at rest, 1 s apart. The first stimulus was always kept at the same intensity (2x threshold intensity for the somatosensory and + 30dB for the auditory modality), while the second stimulus could be one of 9 possible intensities, equally spaced around the intensity of the first stimulation. This meant that participants compared the first central stimulation to either: 4 increasingly lower intensities, 4 increasingly higher intensities or to the same intensity. One second after the second stimulation, a right-ward and left-ward arrow appeared on the screen with the labels “high” and “low”. Participants had to report whether the second stimulation was higher or lower compared to the first stimulus through the pedals. The initial comparison task comprised a total of 72 trials, i.e., 8 for each intensity level. The proportion of “high” responses was calculated for each stimulus intensity and a logistic function was fitted on the data points. The procedure was repeated by spacing the stimuli further apart or narrower until participants reached 0% and 100% of “high” responses for the lowest and highest intensity respectively and stimuli “high” proportions were equally distributed. The detection thresholds at 2% and 98% were estimated and the stimuli were equally spaced along these extremes. Participants underwent the same procedure for somatosensory and auditory stimuli, separately. The two sets of 9 stimuli, for the auditory and somatosensory modality, were then used in the subsequent training and experimental phase (thresholds at 2% and 98% for the somatosensory modality: T02 = 3.15 ± 0.99 mA, T98 = 4.84 ± 1.56 mA; and for the auditory modality: T02 = 88.42 ± 5.78 dB, T98 = 103.61 ± 7.16 dB [mean ± SD]).

After the initial calibration phase, the headset height and the lens focus were adjusted to obtain optimal visual resolution and the correspondence between the real-world controller and the virtual hand was calibrated so that left-ward and right-ward movements were equally comfortable and easy. Participants then underwent a short training phase in VR to familiarise with the experimental task. The training phase consisted of 32 trials, divided equally in *stay* and *move* trials. For each movement type, participants underwent 6 comparisons for the somatosensory modality, 6 comparisons for the auditory modality and 4 control tasks.

After completing the training phase, participants were invited to an adjacent room where the EEG cap was fitted and the electrodes position was digitised through an Eximia neuronavigation system (Nexstim, Helsinki, Finland). The procedure took approximately 5 min to complete, after which the proper experimental phase could begin.

### Experimental design

In each of the 4 experimental runs of approx. 15 min, participants underwent 160 trials, for a total of 640 trials per participant. On top of the virtual table was rendered a fixation cross, centred with the field of view of the camera as well as two indicator circles (distanced ± 0.2 Unity Units [Uu] from the fixation cross and still within the field of view of each eye). Participants were instructed to keep their gaze on the fixation cross and to keep their index finger within one indicator circle or to move it towards the ball located in the circle located in the opposite side of the virtual surface, for the *stay* and *move* conditions respectively.

At the beginning of each run, an arrow indicated the circle in which the participant had to put their index finger. Once the finger was in the circle, the new sequence started. At the beginning of each trial, a virtual ball appeared in the centre of the circle opposite from the participant’s finger. After a delay of 1 s, the fixation cross changed colour for 0.5 s. If the cross flashed green, participants were instructed to move as soon as the cross stopped flashing and to actively reach the ball (*move* condition). If the cross flashed red, volunteers were required to stay still, and the virtual ball reached their resting finger (*stay* condition). As soon as the cross stopped flashing, the ball started moving with a velocity corresponding to the one of any of the previous trials in which a reaching movement was performed. In this way, we could minimise differences in trial time between *stay* and *move* conditions and we could personalise the ball velocity in *stay* conditions according to each participant’s moving pace. If participants moved during a *stay* condition or moved before the cross stopped flashing, a prompt appeared indicating the wrong execution of the trial.

Once participants actively touched (or got touched by) the virtual ball, a somatosensory stimulation could have been administered in 40% of trials, an auditory stimulation in 40% of trials or no stimulation in 20% of trials (*touch* and *audio* each were 256 out of 640 trials while the remaining 128 were control trials).

The intensity of the first – *test* –stimulation (either self-generated or passively received – given upon contact with the virtual ball) was kept constant as the central intensity out of the 9 previously calculated during the calibration phase. Participants were instructed to keep their finger in the same position and one second after the first stimulus, they received a second – *comparison* – stimulation. The number of trials per intensity level was normally distributed so that the number of trials for the comparison of the central intensity against itself was maximised (48 out of 256 trials for the central intensity and 16 out of 256 trials for the lowest or highest intensity).

In control trials, participants received no stimulation as a result of actively touching or passively getting touched by the virtual ball. One second after contact with the ball, the fixation cross in the middle of the screen changed colour (either darker or brighter) for 0.2 s.

Half a second after the administration of the second stimulation (or control task), the virtual ball disappeared and two labels – “high” and “low”– appeared randomly on top of the pedals. Upon pedal press, the selected label increased in size before disappearing. If participants underwent the intensity comparison task (i.e., received a stimulation), they had to report whether the second stimulation was higher or lower in intensity than the first stimulus. If they were administered the control task (i.e., *no-stimulation*), subjects were instructed to answer “high” if the fixation cross flashed brighter or “low” if it flashed darker. The response could be given within a 1.5 s time window. If the timeout was reached, a prompt indicating a missed response appeared on the screen, inviting participants to provide an answer faster on following trials.

**Fig. 1:**
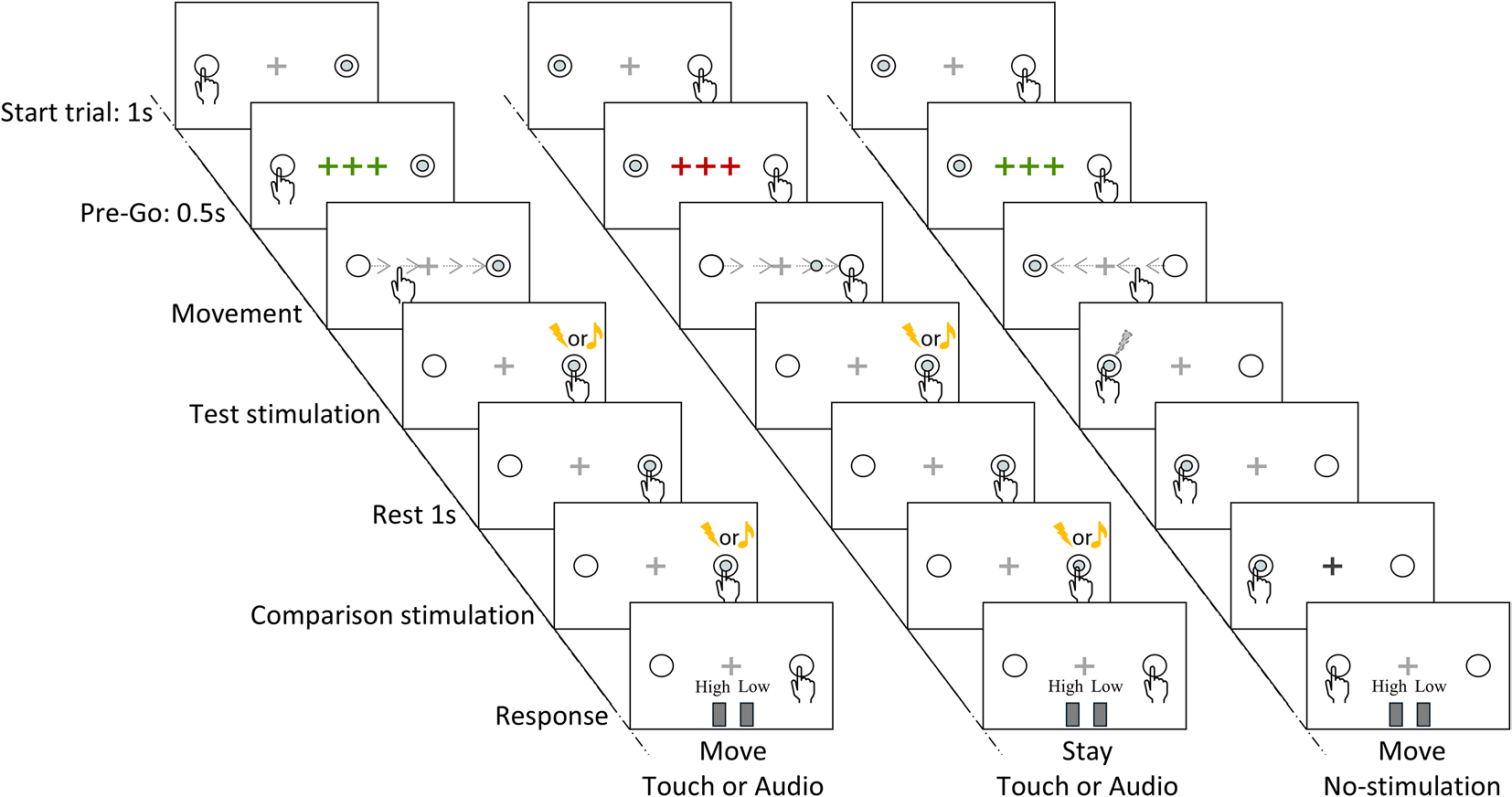
Depiction of an example of a sequence of trials in the experimental paradigm. On the first trial (first column), the participant was instructed to reach and touch the virtual ball positioned in the opposite indicator circle, which administered a first – *test* – stimulation (somatosensory or auditory). After reaching the new position (right indicator circle), the participant was required to stay still and after 1 s a – *comparison* – stimulation of the same modality as the first one was administered. After 0.5 s, participants were required to estimate whether the second stimulation was higher or lower than the first one (comparison task). The next trial (second column) started with the appearance of the virtual ball in the indicator circle opposite to the hand. The participant was instructed not to move and wait for the ball to touch the finger, which resulted in a *test* stimulation (somatosensory or auditory). After the administration of a second stimulation of the same modality, the subject performed the same comparison task. During the following trial (third column), the participant was again instructed to move, but upon contact with the virtual ball, no stimulation was administered. After 1 s the fixation cross flashed darker or brighter and participants were instructed to respond “low” or “high” accordingly (control task). Participants could undergo control trials either in a *move* or *stay* condition.

After the response was registered, a new trial started after a random inter-trial interval between 1.25 and 1.75 s. Subsequent trials always started with the virtual ball appearing in the opposite indicator circle with respect to the participant’s hand. For a depiction of the experimental paradigm, please refer to Fig. 1.

By means of this virtual reality paradigm, participants could always predict the occurrence of the stimulations both during *move* and *stay* trials, since the virtual ball was always visible during active reach or when it was moving towards the resting hand of the participant. This is particularly important since it has been demonstrated that temporal predictions (e.g., predicting the temporal occurrence of a stimulation) could be a confounding aspect in the investigation of sensory attenuation^28,42,55^.

The use of virtual reality also allowed us to deliver a controlled stimulation that was independent and unaffected by the occlusion with an object. Usually, sensory attenuation paradigms require participants to press a button to actively produce a stimulation. This has been argued to be a confounding aspect in the electrophysiological investigation of this phenomenon^56^, since the self-produced stimulus during *motor-and-sensory* trials might mask the tactile and auditory stimulations generated by the button press itself, which are instead fully available during the *motor-only* condition. Since the contact with the virtual ball elicited only the electrical or auditory stimulation when prompted by the experimental paradigm, we were able to eliminate this possible confounding factor associated with pressing a physical button. Moreover, the intensity of the delivered stimulation was independent of the velocity of the reaching movement. Further, spatial attention was balanced across the experiment since right- or left-ward active reaching or ball movements were equally likely during the experiment.

### Behavioural data analysis

Movement velocities are defined as the average velocities during the time that elapsed from the start of the movement (either active hand movement or of the virtual ball) to the contact with the hand. Movement velocities outliers were defined as trials in which participants moved in less than 100 ms and longer than 2500 ms. Differences across conditions were calculated to test whether our velocity personalisation approach was successful and to exclude the possibility that differences in pre-stimulus movement characteristics might have impacted our electrophysiological results. A linear mixed effect model was fitted, having as fixed factors stimulation type (*touch*, *audio* and *no-stimulation*) and movement type (*move* or *stay*) and as random intercept the subjects.

Since the intensity comparison task and the control task were substantially different, we kept the behavioural analyses separate for the calculation of response times and accuracy. Response time was defined as the time participants took to give an answer from the moment when the labels “high” and “low” appeared on top of the virtual pedals. Accuracies for the intensity comparison task were the percentage of correct comparisons (e.g., answered “low” when the second stimulus had a lower intensity than the first one) over the total number of possible comparisons (i.e., all intensities except for the middle intensity). Accuracies for the control tasks were simply the proportion of correct responses (according to the instructions that participants received) over the total possible number of control responses.

Two linear mixed effect models were fitted over response times and accuracies for the intensity comparison task, which comprised as factors stimulation type (*touch* and *audio*) and movement type (*move* or *stay*). Identical models were calculated using response times and accuracies for the control task, which had as factor movement type (*move* or *stay*). All models were fitted with a random intercept by participant.

Lastly, to determine whether the perception of the first stimulation was affected by self-producing or passively perceiving it, we first calculated the proportion of trials in which the participants reported that the second stimulation was higher than the first one. For each participant, movement and stimulation condition, we fitted logistic psychometric functions on the proportion of “high” responses using the Psignifit Matlab toolbox^57^. This allowed to determine the point of subjective equality (PSE) across conditions, i.e., the stimulus intensity at which the participants would judge the test and comparison stimulus to be the same intensity in 50% of trials. PSE during *move* and *stay* trials were compared by fitting linear mixed effect models separately for the auditory and tactile modality. We expected the PSEs for self-produced stimuli to be significantly lower than the ones for externally-perceived stimulations, indicating that participants perceived the self-generated stimuli as lower in intensity than the same stimuli administered at rest.

### EEG data collection and preprocessing

Data were collected using a 64-channel active electrode EEG system (ActiveTwo, BioSemi, Amsterdam, Netherlands) at a sampling rate of 2048 Hz, with head electrodes placed in accordance with the extended 10–20 system. Preprocessing of the EEG data was performed using SPM12^58^, FieldTrip^59^ and in-house MATLAB scripts. We first visually identified and interpolated bad channels (5 ± 3 [mean ± SD]), then the continuous EEG recording for each participant was re-referenced against the average reference, down-sampled to 512 Hz and high-pass filtered (0.1 Hz, firws, one-pass zero-phase, -6 dB cut-off). Then, we corrected for eye-blinks and horizontal eye movements through a topographical confound approach^60,61^. Next, epochs were defined as windows spacing from − 2 s to 2 s around ball touch (i.e., when the first stimulus was presented or not) and a low pass filter was applied (45 Hz, firws, one-pass zero-phase, -6 dB cut-off). Epoched datasets were then visually inspected and bad data segments were marked and excluded. Each dataset was further denoised using Denoising Source Separation (DSS)^62–64^ to maximise the reproducibility of stimulus evoked activity in response to tactile, auditory and control conditions either during self-produced or externally-perceived trials. DSS is a semi-blind source separation technique that uses the time-locked electrophysiological activity to unmix the continuous recording into sources that gradually explain the time-locked signal. To determine the (cumulative) number of sources to retain, we calculated the log ratio between each experimental condition and the relative control condition, across channels and time-points before stimulation and after 500 ms. We then kept the number of components that minimised the differences between stimulation trials (*audio* or *touch*) and controls (*no-stimulation*). A total of 7 components for each subject were maintained. DSS therefore allowed us to denoise our signal to further prevent that differences between the recorded electrophysiological activity across conditions might impact our results. EEG data was then baseline corrected using a pre-stimulus window ranging from − 50 to − 5 ms.

To ensure that the data were equivalent between the EEG and the behavioural analyses, in both datasets we kept only trials that (i) did not contain any movement velocity outlier, (ii) did not have any missed response and (iii) were clean EEG trials. On average, we excluded a total of 13.90% of trials (SD = 4.73%). 0.84% (SD = 1.18%) of the total trials were movement velocity outliers, 4.08 (SD = 3.25%) were missed responses and 9.90% (SD = 3.14%) contained artefactual trials, while 0.92% were shared. After exclusion, out of 640 trials we obtained on average 551 (SD = 31) trials. Lastly, out of the initial 29 participants, one participant was excluded because they couldn’t perform the task. The following results are therefore computed on 28 participants.

### EEG data analysis–qualitative assessment

It has been previously argued that a possible confound in the investigation of sensory attenuation is the violation of the assumption that the potential recorded during *motor-only* conditions is the same in *motor-and-sensory* trials. In our previous study^39^ we excluded this possibility by investigating the pre-stimulus interval and pointing out that the difference between the motor potential recorded upon stimulus administration and when no stimulation was given resulted zero, strongly suggesting that the electrophysiological motor activity in the two conditions was compatible. Before reporting the formal statistical analyses, in the present work, we qualitatively assessed the time-locked electrophysiological activity to (i) verify that the tactile and auditory stimulation resulted in prototypical Somatosensory Evoked Potentials (SEP) and Auditory Evoked Potentials (AEP) and – importantly – to (ii) confirm that the measured electrophysiological activity in the pre-stimulus interval for *move* and *stay* trials (either before a stimulation or not) was similar.

### EEG data analysis–sensory attenuation in somatosensory and auditory modalities

Main analyses were performed according to the SPM framework for M/EEG analysis. This required the epoched electrophysiological data to be converted into a 3D image (scalp space x intra-trial samples). To achieve this, each subjective electrode localisation map was first projected onto a 2D space and re-scaled into a 32 × 32 mask, so that the position of the electrodes on the mask corresponded to the location of the sensors on the scalp. EEG data from each channel was then entered into the corresponding location on the mask and then linearly interpolated for each sample^65^. This procedure allowed to interpolate EEG 2D data arrays into 3D images to be further analysed with correction for multiple comparisons using random field theory^65,66^.

Since we had a priori hypotheses about the electrophysiological responses evoked by the tactile or auditory stimulation, we selected a window spanning from − 50 to 500 ms around ball-touch (i.e., the first stimulation, either self- or externally-produced). In this way, we obtained one 3D image of dimensions 32 × 32 × 283 (scalp space x intra-trial samples) for each trial. Then, all images were inserted into a first-level multiple regression model, which had one dummy regressor for each possible condition combination (6 in total). After model estimation, we obtained a 3D β estimate for each condition with the same dimensionality as the initial images, which corresponded to the averaged time-locked potential of each condition, for each participant.

In the field of sensory attenuation, to test whether a self-produced stimulation evoked potential is attenuated compared to an externally perceived sensation, a control condition is required. In classical contingent paradigms, a movement-and-touch condition is subtracted of a movement-only condition before being compared to a touch-only condition^26^. In this way, any potential evoked by the movement alone can be accounted for, and only the potentials elicited by the stimulation (either self-generated or externally perceived) can be compared. In a similar fashion, our study allowed for the recording of two control conditions, i.e., conditions in which no stimulation was administered when actively reaching or passively being touched by the virtual ball. The additional advantage of our approach was that we could control not only movement-related evoked potentials, but also potentials evoked at rest, possibly due to stimulus expectation or attentional demands.

To directly test whether the potential evoked by the self-elicited stimulation was attenuated compared to the one evoked by the externally-generated stimulation – while controlling for potentials elicited by movement or stimulus expectation – we implemented two mass-univariate multiple regression analysis for each stimulation modality. Each model included one regressor for each condition of interest, as well as for each subject. Mean differences across conditions were tested via F-tests and therefore, in its interpretation, both models were equivalent to a 2 × 2 ANOVA, having as factors: stimulation (*touch* or *audio* and *no-stimulation*) and movement type (*move* or *stay*). All analyses were performed with a cluster-forming threshold of *p* < 0.001; only clusters withstanding at the cluster-level with family-wise error (FWE) corrected threshold of p_FWE_ <0.05^67^ are reported here.

Usually, in contingent paradigms, a *motor-only* condition is subtracted from a *motor-and-sensory* condition to obtain a corrected potential of self-produced stimulus perception to be compared against a *sensory-only* condition. In a similar fashion, in the present study we tested differences between self-generated and externally-produced sensations, while controlling for a compatible control *no-stimulation* condition, by selectively exploring the interaction term across conditions, in each model independently (i.e., [*touch move* – *touch stay* – *no-stimulation move* + *no-stimulation stay*] and [*audio move* – *audio stay* – *no-stimulation move* + *no-stimulation stay*]).

### Control analysis–second stimulation

To further assess the specificity of sensory attenuation, we also tested for electrophysiological attenuations upon administration of the second stimulation. We theorised to find no interaction effect between stimulation and movement type, excluding the presence of a sensory attenuation effect for the second stimulation and further substantiate the idea that our main results are not indeed based on the interaction between each movement and stimulation condition.

To test this hypothesis, we first defined epochs around the second stimulation, ranging from − 50 to 500 ms and we baseline corrected the data using a window from − 50 to -5 ms before the stimulation. We then fitted models identical to the main analyses at the first and second level, separately for the tactile and auditory modalities. Identically to the main analyses, mean differences across conditions were tested via F-tests and therefore, in its interpretation, both models were equivalent to a 2 × 2 ANOVA, having as factors: stimulation (*touch* or *audio* and *no-stimulation*) and movement type (*move* or *stay*). All analyses were performed with a cluster-forming threshold of *p* < 0.001. We reported the clusters surviving FWE correction with a threshold of p_FWE_ <0.05.

### Correlation between electrophysiological and behavioural indices of sensory attenuation

To formally test whether behavioural attenuations for self-produced sensations were associated with electrophysiological indices of suppression, we performed a correlation analysis. First, for each subject and each sensory modality independently, we obtained a linear combination of the β estimates which resulted by subtracting the average of the *move no-stimulation* and *stay stimulation* trials to the average of the *move stimulation* and *stay no-stimulation* trials. These are also commonly referred to as contrast images. Positive differences in early-latency components (N100 or N1 for the somatosensory and auditory domain) or negative differences in mid-latency components (P200 or P2 for somatosensory and auditory modalities respectively), therefore, indicated an electrophysiological attenuation for self-produced compared to externally-generated stimulations (i.e., sensory attenuation).

Similarly, for the behavioural domain, we calculated for each participant the difference between the PSE in the *move* and *stay* conditions. A negative difference would indicate that PSEs for self-generated stimuli were lower than externally perceived ones, i.e., stimuli resulting from *move* trials were perceived as less intense.

The contrast images for each subject were then inserted into a second level mass-univariate multiple regression analysis, having as only regressor the behavioural index of attenuation. In its interpretation, the model is equivalent to a Pearson correlation between electrophysiological and behavioural indices of attenuation. Positive and negative correlations were then tested via t-statistics.

## Results

### Behavioural results

**Fig. 2:**
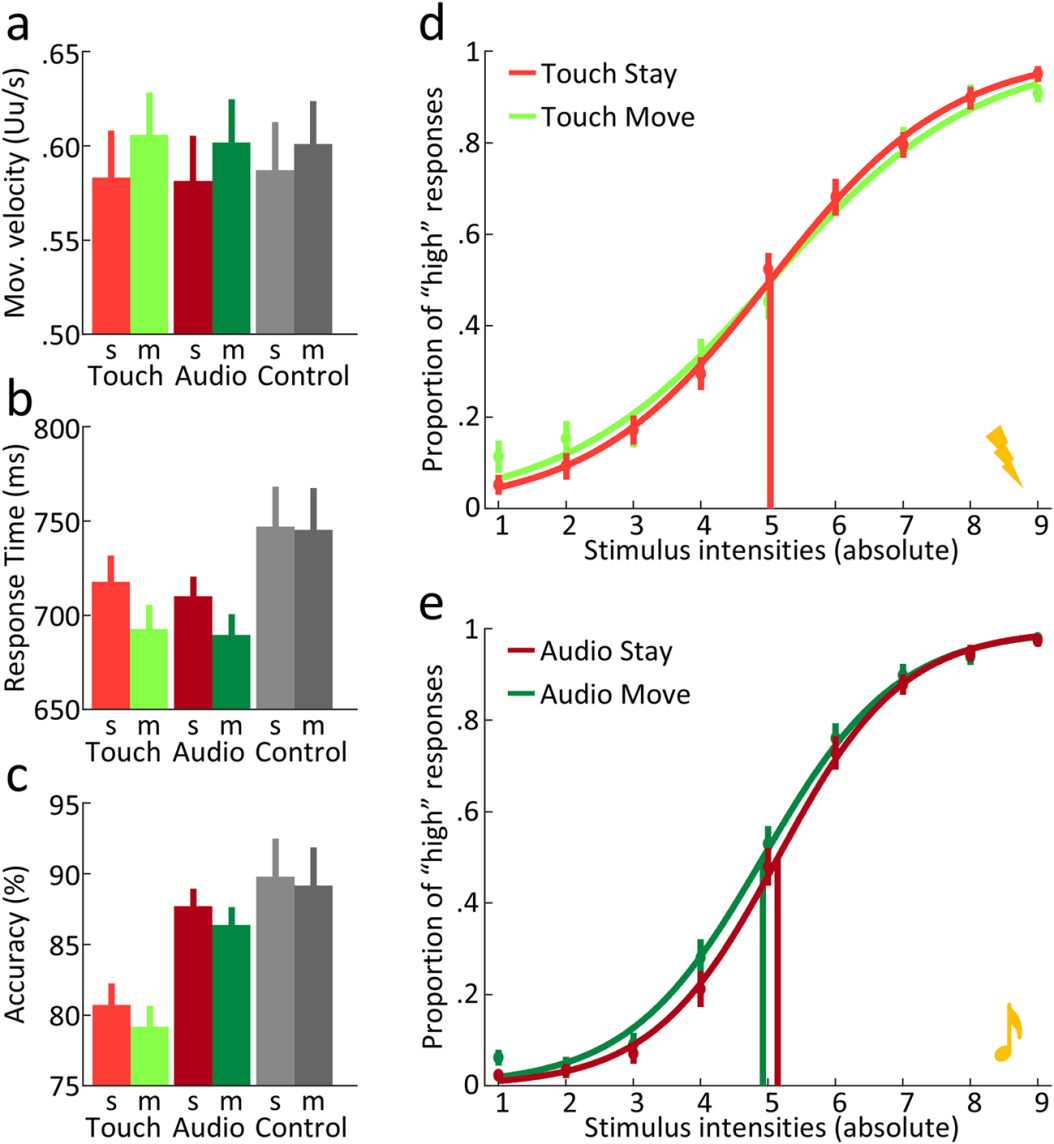
Behavioural results. (**a**) Movement velocity, (**b**) response time and (**c**) accuracy across movement type (*move* or *stay*) and stimulation conditions (*touch*, *audio* or *no-stimulation*). (**d, e**) Psychometric functions fitted over the average “high” response proportion for each stimulus intensity (absolute) and divided for movement conditions and stimulation type. Vertical lines originating from the psychometric functions represent PSEs. Error bars in all graphs represent standard error. s = *stay*, m = *move*.

Movement velocities were different across *move* and *stay* trials (ƞ^2^ = 0.090, F_1,162_ = 15.936, *p* < 0.001) but did not differ across stimulation type (ƞ^2^ = 0.002, F_2,162_ = 0.140, *p* = 0.869) and their interaction was not significant (ƞ^2^ = 0.004, F_2,162_ = 0.327, *p* = 0.721). Move trials were overall 0.019 Uu/s (circa 11.19 ms) faster than *stay* trials (see Fig. 2a). Although this indicates that our velocity personalisation approach was not optimal, we consider it very unlikely that this limitation significantly influenced the subsequent results. If the different trial lengths led to a difference between the electrophysiological correlates of *stay* and *move* conditions, it would have affected equally both the stimulation (*audio* or *touch*) and the respective control *no-stimulation* trials, thus cancelling out in the subsequent analyses.

Response times for the comparison task were faster in *move* than in *stay* trials of 22.83 ms (ƞ^2^ = 0.105, F_1,108_ = 12.605, *p* < 0.001) but did not differ across *touch* or *audio* stimulations (ƞ^2^ = 0.006, F_1,108_ = 0.661, *p* = 0.418). The interaction between the two factors was also not significant (ƞ^2^ = 0.001, F_1,108_ = 0.121, *p* = 0.728). Response times for the control task instead showed no differences between *stay* and *move* conditions (ƞ^2^ = 0.001, F_1,54_ = 0.051, *p* = 0.821). See Fig. 2b.

Lastly, participants were more accurate on the auditory task compared to the tactile task (ƞ^2^ = 0.166, F_1,108_ = 21.488, *p* < 0.001) of 7.08% on average. Accuracy did not differ across movement conditions (ƞ^2^ = 0.010, F_1,108_ = 1.057, *p* = 0.306), nor was the interaction between the two factors significant (ƞ^2^ = 0.000, F_1,108_ = 0.010, *p* = 0.920). Accuracy for the control task also did not differ across *move* or *stay* trials (ƞ^2^ = 0.010, F_1,54_ = 0.571, *p* = 0.453). For illustration, see Fig. 2c.

Lastly, PSE for the auditory modality were significantly lower in the *move* than in the *stay* condition (ƞ^2^ = 0.106, F_1,54_ = 6.38, *p* = 0.014). For the tactile modality we observed no differences across movement types (ƞ^2^ = 0.000, F_1,54_ = 0.010, *p* = 0.921). For a depiction, see Fig. 2d and *e*.

### Electrophysiological results–qualitative evaluation

**Fig. 3:**
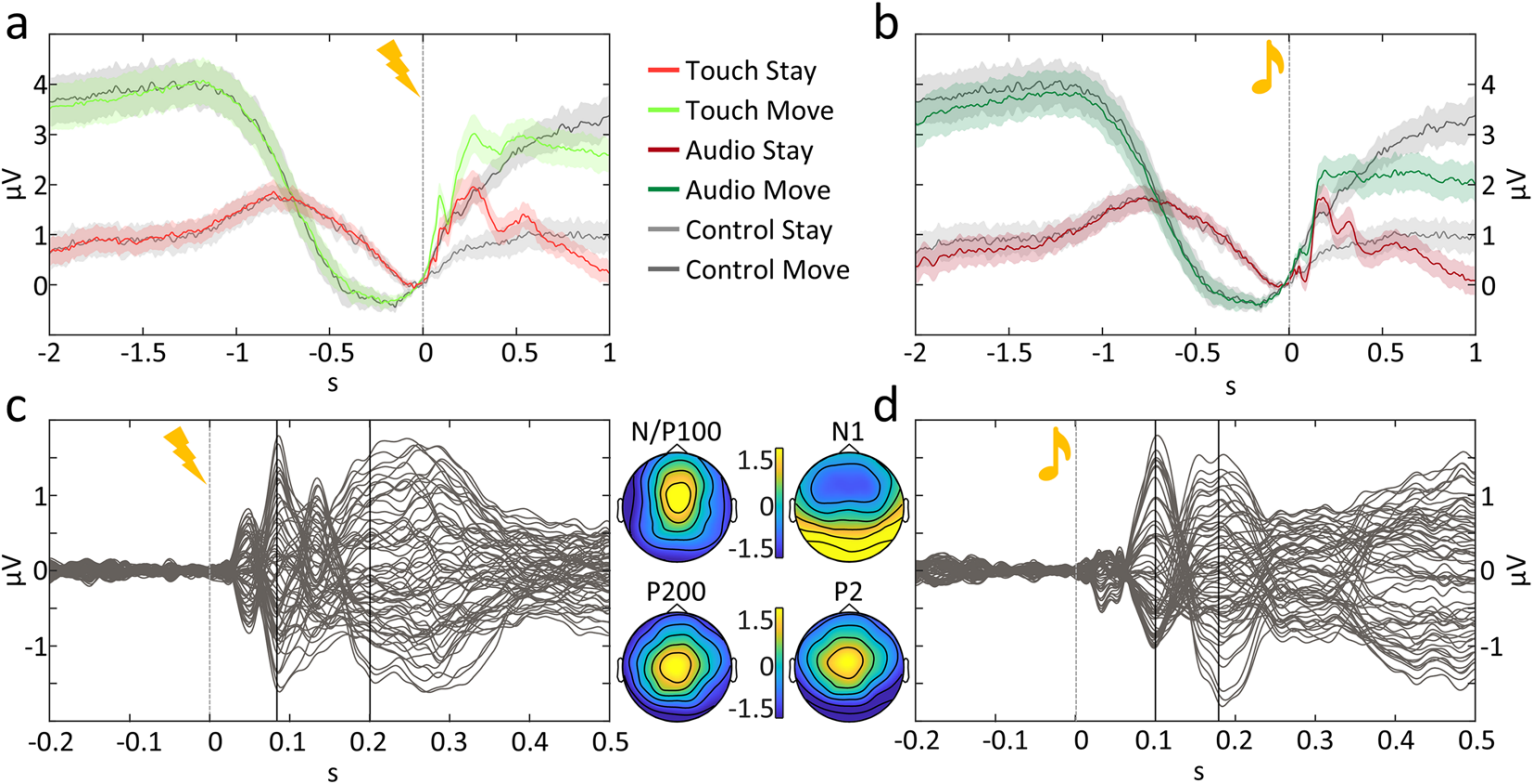
Qualitative analysis of potentials evoked by stimulus onset. (**a, b**) ERPs for each condition for a subset of centro-lateral electrodes, divided for tactile and auditory stimulation in comparison to the control (*no-stimulation*) trials. (**c, d**) Superimposed plot of all electrodes (butterfly plot) of the averaged differences between *touch* or *audio* trials and *no-stimulation* trials matched by movement type. All ERP images are plotted for a cluster of central electrodes (FC3, FC1, C1, C3, CP3, CP1, P1, P3, Pz, CPz, FC2, FCz, Cz, C2, CP2, P2). On the side, scalp distributions of the difference between *touch* or *audio* and *no-stimulation* trials at the time points marked in the respective butterfly plots. In all panels, dotted lines at 0 s represent the moment at which the stimulation was administered or the control (*no-stimulation*) condition was time-locked.

The averaged electrophysiological activity for the movement condition in all sensory modalities (either *touch*, *audio* or *no-stimulation*) was characterised by an increasing pre-stimulus negativity that gradually increased before stimulus onset. This is likely due to stimulus anticipation^68–70^, motor preparatory processes^71,72^ and motor execution^73–75^. In *stay* conditions, across sensory modalities, the pre-stimulus activity was also characterised by a negativity, possibly reflecting a process of stimulus anticipation (see Fig. 3a and *b*). Differences between movement conditions were accounted for in our analyses by the inclusion of a control (*no-stimulation*) condition, for both *move* and *stay* trials. As mentioned before, the robustness of our results relies on the assumption that the measured electrophysiological activity across stimulation types is equivalent within *move* and *stay* trials. In support of this hypothesis, the pre-stimulus activity of the main effect of electrical or auditory stimulation (*touch* or *audio* – *no-stimulation*) averages around zero, giving aid to the assumption that – indeed – the measured electrophysiological activity across *move* or *stay* trials was equivalent.

From tactile onset, our paradigm elicited a typical SEP. Figure 3c shows the difference between the average *touch* trials across participants subtracted of the control (*no-stimulation*) trials across electrodes with the expected evoked potentials, i.e., P50, N/P100 and P200 resulting from electrical stimulation of the right index finger. The corresponding topographic maps (Fig. 3c, right) confirm a slight left lateralized negative voltage distribution of the somatosensory evoked potential (SEP) components on the scalp (N/P100).

The auditory stimulation also elicited a typical AEP. As can be observed in Fig. 3d, the difference between *audio* trials and *no-stimulation* trials elicited prototypical potentials, i.e., N1 and P2.

### Electrophysiological results – sensory attenuation

To test for differences in the electrophysiological activity evoked by either self-producing or passively attending a stimulation (i.e., first stimulus), while controlling for the effects of a control condition, we investigated the interaction term between movement and stimulation type.

**Fig. 4:**
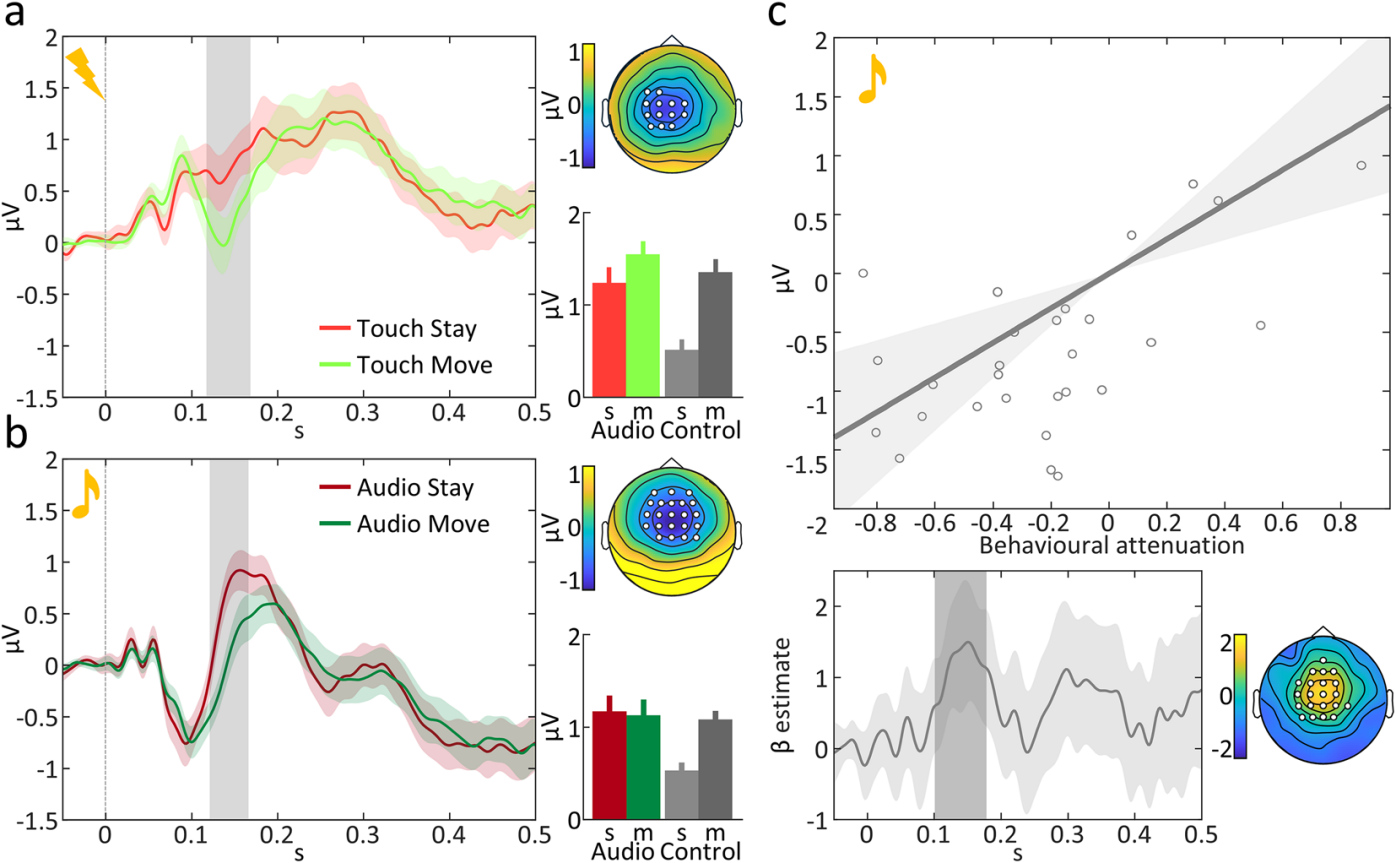
Electrophysiological results. Interaction effect stimulation x movement for (**a**) the tactile modality and (**b**) the auditory modality. Both panels show the ERP plot of the subtraction of each stimulation condition to the corresponding *no-stimulation* condition. The average of the electrodes comprising the cluster are plotted. Gray shaded areas represent significant time points with p_FWE_ < 0.05 and line contours are standard errors. Dotted lines at 0 s represent the moment at which the auditory or somatosensory stimulation (or *no-stimulation*) was administered. Scalp distributions represent the difference between ERP plots across the significant time window. Bar-plots show the values of each condition across the significant time window, with standard errors. (**c**) At the top, correlation between behavioural and electrophysiological measures in the auditory comparison task. On the x-axis is the difference between PSE across subjects, while on the y-axis the contrast image (*audio move* – *audio stay* – *no-stimulation move* – *no-stimulation stay*) is plotted, averaged over electrodes and timepoints as revealed by the whole-brain correlation analysis. The slope of the correlation line is the average β estimate from the GLM. Grey shaded areas are confidence intervals. At the bottom left, the time-course of the β estimate plotted for the significant cluster. Gray shaded area is the confidence interval while the darker shaded area is the time of significance. On the right, scalp distribution of the β estimate across the significant time-window. Highlighted electrodes are the electrodes included in the cluster at any time-point. s = *stay*, m = *move*.

For the somatosensory modality, one cluster survived cluster-level FWE correction in central electrodes and starting at 0.117 s to 0.168 s post stimulus (peak at 0.145 s, centroid: CP1, ƞ^2^ = 0.229, F_1,81_ = 24.11, p_FWE_ = 0.010). This positive potential (P200) was reduced in amplitude when the stimulation resulted as a consequence of a movement compared to the corresponding potential administered at rest (Fig. 4a).

For the auditory modality, one cluster reached significance, located in centro-frontal electrodes and starting at 0.121 s to 0.166 s post stimulus (peak at 0.137 s, centroid: FC4, ƞ^2^ = 0.331, F_1,81_ = 40.04, p_FWE_ < 0.001). This positive potential (P2) was suppressed for self- compared to other-generated auditory stimulations, when controlling for *no-stimulation* conditions (Fig. 4b).

Notably, concomitant to the fronto-central positivity, our analyses also revealed an occipital negativity that survived cluster-level FWE correction (peak at 0.135 s, centroid: O2, ƞ^2^ = 0.303, F_1,81_ = 35.25, p_FWE_ = 0.001). This is consistent with a dipolar scalp distribution and, since typically auditory evoked potentials around 200 ms are reported in fronto-central electrodes^27,28^, this cluster was not further analysed.

### Electrophysiological results – correlation between electrophysiological and behavioural attenuation indices

To formally test whether behavioural indicators of sensory attenuation were related to electrophysiological suppressions, we performed a correlation analysis on the previously defined − 50 to 500 ms window (first stimulus) and throughout electrodes. Results for the auditory modality revealed that one central cluster, peaking at 0.156 s (and ranging from 0.102 s to 0.178 s) was positively correlating with behavioural indices of attenuation (centroid: C2, ƞ^2^ = 0.529, T_27_ = 5.51, p_FWE_ = 0.001). We identified this peak corresponding to the previously identified auditory P2. The present results therefore indicate that at larger auditory P2 suppressions corresponded larger PSE reductions for self-generated stimulations (for a depiction, see Fig. 4c).

The correlation analysis for the somatosensory modality revealed no significant clusters.

### Electrophysiological results–control analysis

To further corroborate our electrophysiological results, we performed a control analysis by investigating possible interaction effects for the potentials evoked by the second – *comparison* – stimulation. We expected at this timepoint no sensory attenuation effect since the stimuli administered after the first – *test* – stimulation (that could be either self- or externally-generated) were perceived at rest. If our analyses had pointed out any kind of interaction, this could have indicated that our results might also be explained by possible interactions between movement and the stimulation, i.e., the motor (or non-motor) potential across stimulation conditions is not equal. As expected, our control analysis on the second – *comparison* – stimulus revealed no interactions between movement type and stimulation.

## Discussion

Despite the extensive literature investigating sensory attenuation, it remains unclear whether behavioural attenuation of self-generated stimuli reflects electrophysiological suppression. In the present experiment, we employed a previously validated VR paradigm that allowed for more comprehensive control of stimulus properties, attentional requirements, and other predictability factors during an intensity comparison task, alongside concomitant EEG recording. In this way, we were able to probe behavioural and electrophysiological correlates of sensory attenuation both, in the somatosensory and auditory modalities.

Behavioural analyses revealed that participants perceived the self-generated stimulations as less intense only in the auditory modality. This was accompanied by higher accuracy in the auditory comparison task than in the somatosensory one. The intensity comparison task in both sensory modalities, however, showed a faster response time for *move* than *stay* trials.

At the electrophysiological domain, the P200 for somatosensory and the P2 for auditory stimulations were attenuated when the stimulations were self-generated compared to when they were passively administered, while controlling for *move* and *stay* conditions where no stimulation was administered. We found no evidence of attenuation for the second stimulation. Lastly, the correlation analysis revealed that the suppression at the auditory P2 positively correlated with the decrease in perceived intensity as indexed by the intensity comparison task.

### Behavioural measures of sensory attenuation

Contrary to our expectations, we found evidence of behavioural sensory attenuation in the auditory domain, but not in the tactile modality. It is usually assumed that our brain uses an efference copy of a motor command to predict motor outcomes and to estimate whether the effective sensory feedbacks generated by motor plans coincide with the predicted ones^50^. The consequence of this prediction is often believed to be an attenuation of action outcomes, i.e., already predicted or redundant information is kept away from our senses to leave space for more information-rich afferents. This concept is supported by numerous studies demonstrating that self-generated sensory outcomes are perceived as less intense compared to identical stimulations administered at rest^8,17^ or that congruent action-effects are suppressed rather than incongruent ones^9,20^. Moreover, these findings received support across different sensory modalities, such as auditory^16–18,25,76,77^, visual^9,78,79^ and tactile^7,8,12,20^ substantiating this concept even more. However, the current state of the art suggests a more complex picture, as several studies indicate that motor efferents might adapt perception according to the different sensorial context or task requirement, rather than simply dampening it. For instance, Myers and colleagues^24^ reported over a series of experiments that perceptual acuity is enhanced when actively generating a supra-threshold auditory stimulation during an intensity comparison task. Moreover, Reznik and colleagues^16^ reported enhanced perceived loudness when the self-generated stimuli were at near-threshold level, in accordance to other studies that used similar stimuli intensities^24,47^, while self-generated supra-threshold auditory stimulations were attenuated compared to passively perceived identical stimuli. This concept is further corroborated by studies utilizing a similar procedure and stimulus modality^77^ and by evidence of larger suppression effects for greater stimulus intensities, although in the tactile domain^80^. In other words, self-generated sensations might be weighed differently based on the nature of the task at hand. If the task requires a higher precision, sensations that are self-produced might be enhanced rather than suppressed. The argument that predictable effects of a voluntary actions are not automatically suppressed is further supported by studies that did not point out attenuations for self-generated stimulations^21,22^.

Concerning the present study, since both auditory and somatosensory modalities were probed through an identical comparison task, it is unlikely that the absence of an attenuation effect in the somatosensory domain is to be attributed to the task itself. Additionally, stimuli intensities were calculated through identical calibration procedures in both sensory modalities, so that the highest and lowest stimulation intensities corresponded to the 98% and 2% detection accuracy during the calibration phase. Moreover, both stimuli were square waves of 0.2 ms duration. In other words, if our comparison task was sufficient to induce a shift in perceptual threshold in the auditory modality, one would expect the same to happen for somatosensory stimuli. The reason behind this discrepancy, therefore, requires further investigation. One possibility is that sensory attenuation operates differently across senses, at least when conscious perception is involved. It is possible that, given the tight coupling of somatosensation with the body and the motor system, somatosensory stimuli are weighed differently by predictive mechanisms as compared to other senses^81^. This might be especially true in an ecological scenario such as our VR setup. It is in fact well known that our every day’s sensory-motor interactions are mostly based on visuo-haptic integrations and it is plausible that they hold an cardinal role in human perception^82^. Furthermore, it has also been demonstrated that somatosensory stimuli administered during or before movement are “gated”, i.e., they are physiologically attenuated from sensory reafference^83,84^. This motor-related inhibition is maximal at the limb that is being moved^85^ and it extends over time after movement initiation^86,87^. Since movement execution in similar tasks has not been demonstrated to affect auditory perception^88^, it is possible that in our paradigm gating phenomena might have affected the perception of the self-generated tactile stimulation – at least concerning their subjective perceived intensity – thereby explaining the absence of sensory attenuation at the behavioural level in the somatosensory modality.

Lastly, one possibility that could reconcile our behavioural findings is that the comparison task in the somatosensory modality was of greater hindrance. This is not only indexed by higher variances in the hit rates, but also by a significant overall lower accuracy obtained in the tactile comparison task compared to the auditory modality. Although speculatively, the greater precision required by the task might have prevented participants from experiencing a univalent effect, as a consequence of self-producing sensations. This hypothesis might be supported by recent findings that utilised a comparison task across two different sensory modalities (visual and auditory) in which an enhancement effect at the behavioural level has been pointed out for the visual modality, that had lower accuracies^15^. In other words, when the accuracy for the comparison task was even lower (possibly indicating a greater difficulty at the task) participants perceived self-generated stimuli as enhanced rather than attenuated. Although previous behavioural studies investigating sensory attenuation in the somatosensory domain reported reduced intensity perceptions for self-generated stimulations^5,7,8,19,48^, participants were required to judge forces, strokes or haptics. In the present study, to utilise a stimulation that was compatible for both behavioural and electrophysiological measurements, we opted for electrical pulses. It is also possible that not only the sensory domain (auditory or tactile), but also the stimulation administration modality (force or electric pulse) can differently probe our perceptual abilities under self- or external-generation. Further studies will be required to substantiate these hypotheses.

Lastly, our findings also revealed a decreased response time at the comparison task when the stimulation to be compared was self-generated, rather than when it was externally administered, across sensory modalities. It has been previously demonstrated that predictable events are linked to decreased response times^89–92^. It is plausible that the perception of self-generated stimuli, being subject to qualitatively different predictive processes, might have been facilitated; thus, the following response at the comparison task was administered faster.

### Electrophysiological measures of sensory attenuation

The electrophysiological results of attenuation for auditory P2 and somatosensory P200 are consistent across senses. Interestingly, our findings did not indicate attenuation of earlier ERP components, which is typically a common result in sensory attenuation, both in the auditory^6,14,27–34^ and, despite scarce, somatosensory domain^39–41^. One possibility is that participants were required to estimate the intensity of the second stimulation – which had a varying intensity – to the intensity of a first self- or externally-produced stimulation, which had always the same intensity. Other studies that investigated sensory attenuation through comparison tasks required participants to indicate which one of the two stimulations was more intense rather than prompting a unilateral comparison^14,15^. Results from Ody and colleagues^14^ showed an attenuation for auditory N1 and for visual N1 and P2 when the stimulation was actively produced in comparison to when it was passively generated. Similar findings were also replicated by another study from the same group^15^. Early ERP components have been demonstrated to be influenced by attentional factors, both in the auditory^93–95^ and tactile domain^96–98^. Also, attentional factors have been demonstrated to influence the auditory N1 suppression that characterizes self-generated stimulations^28^. It is possible therefore that participants did not pay attention to the first stimulation as it was completely predictable both in its temporal and identity characteristics, resulting in an overall dampening of earlier electrophysiological components.

On an additional note, the present electrophysiological results were obtained through a virtual reality setup validated in a previous study, which allowed us to control for other possible confounding effects that might impact on the attenuation for self-generated movements. More specifically, the experiment was designed to minimize the influence of stimulus properties, spatial attention and temporal expectation (see also Methods and^39)^. It is unlikely, therefore, that our results might be influenced by these aspects. Further, similarly to previous studies that controlled attentional requirements by asking participants to undergo interfering tasks such as counting the number of stimulations^28,34^ or estimating the time interval between consecutive stimulations^99^, in the present study volunteers needed to attend stimuli independently of whether they were presented in the *move* or *stay* conditions to make an intensity judgment. Additionally, immersive VR settings might lead to higher levels of concentration when performing a task^100,101^, possibly reducing the likelihood that attention differences across conditions might explain the reported electrophysiological differences.

Since participants were administered a second somatosensory or auditory stimulation at rest, we could further verify that our main analyses were not influenced by interactions between movement type and stimulation other than the sensory attenuation effect. The absence of any interaction effect at the second stimulation further corroborates this concept, i.e., the electrophysiological correlates of the motor (and non-motor) activity are identical across *touch* or *audio* and *no-stimulation* conditions.

### Bridging behavioural and electrophysiological suppression

Lastly, our findings indicated a positive correlation between the auditory P2 suppression and the perceived reduced intensity for self-generated sensations. Contrary to our expectations, however, we did not find the same relation in the somatosensory modality. Similar contrasting findings across sensory modalities were also observed by another study by Ody and colleagues^14^. In their experiment – which was until now the only other study that formally tested a correlation between behavioural and electrophysiological suppressions – the authors reported an electrophysiological attenuation for self-generated sensations both in the auditory N1 and visual N1 and P2 components, but perceptual PSEs in both sensory modalities did not show a difference between active and passive conditions. Lastly, behavioural attenuation only in the visual task was correlating with visual P2 attenuation. Although in a different sensory modality, we replicate the previous findings obtained by Ody and colleagues, which suggest that the electrophysiological component underlying the subjective intensity reduction is a mid-latency ERP. Although the authors reported a correlation between behavioural and electrophysiological attenuations despite no evidence of behavioural suppression, it is still possible that in the present study the lack of a behavioural effect in the somatosensory domain (for the reasons discussed above) have also rendered the correlation with the electrophysiological suppression insignificant.

The P2 for auditory stimuli has been demonstrated to be modulated by stimulus intensity^102–104^, therefore it is plausible that attenuations of this component produced by self-generation of a stimulation might be responsible for the subjective reduction of perceived intensity of the same stimuli. Moreover, components within the same time-windows have also been theorised to be involved in selfhood attributions. For instance, Ghio and colleagues^105^ found auditory N1 attenuation both for action observation and execution. However, P2 attenuations were stronger self-produced stimuli compared to observed tone production, suggesting that the P2 might be the electrophysiological correlate of authorship over the consequences of one’s own actions (also defined as the sense of agency). Similar conclusions were also advanced by Timm and collaborators^106^ who reported that N1 suppressions happened irrespective of agency conditions, while P2 attenuations correlated with agency reports from participants. Sensory attenuation has often been acclaimed as one of the mechanisms that our brain utilizes to distinguish oneself from others^1,2^ and it has been often considered as an implicit measure of the self of agency^43,107^. This interpretation might be consistent with our results, although it has not been formally tested in the present work.

Therefore, N1 and P2 suppressions for self-generated stimulations are possibly two functionally distinct phenomena^108^ which, more often than not, are existing as two consecutive steps. For instance, cerebellar patients exhibit an auditory N1 suppression for self-generated stimulations, but not P2^109,110^. Mid-latency components seem also to be more resilient towards attention modulations^28,99^ and could be overall a more direct measure of sensory-specific predictions^44,111^.

Alternatively, it is also possible that the electrophysiological suppression and perceived intensity reduction for self-produced stimuli are two distinct phenomena^44^. This conclusion might explain why, although electrophysiological studies almost unanimously report early and mid-latency ERP suppression, studies using behavioural measures of sensory attenuation are often mixed, with some reporting attenuation effects^5,7,8,12–19^ while others reporting enhancements in stimulus intensity perception^16,21,23–25^. It is possible therefore that our results of positive correlation between auditory P200 and perceived intensity reduction only capture some shared variability in the relationship between behavioural and electrophysiological counterparts of sensory attenuation.

Additionally, distinct ERP components may carry behavioural relevance in different sensory domains, reflecting modality dependent implementations of the same underlying mechanisms. This might be due not only to differences in the temporal domain in which sensory systems operate^112,113^ but also to the relevance of the sensory modality to the task itself^114^. In contrast to the study by Ody and colleagues^14^, in the present paradigm, participants were immersed in a virtual scenario where they were required to attend visuo-tactile and visuo-auditory contingencies (*touch* or *audio* conditions), which have been theorised to be processed differently by our sensorium^115^. This perceptual difference driven by task requirements and our setup might be responsible to the modality specificity of our results and of the results obtained by Ody and colleagues^14^. Further research is needed to further explore whether the electrophysiological-behavioural coupling reported here and in previous studies is indeed a by-product of the paradigm itself or is an intrinsic characteristic of sensory attenuation.

In short, we were able to collect behavioural and electrophysiological measures of sensory attenuation for the somatosensory and auditory modality in a unique experimental setup and utilising a stimulation that was consistent in both senses and throughout EEG and behavioural measures. Despite consistent electrophysiological attenuation across sensory modalities, we report sensory attenuation at the behavioural level only in the auditory modality. Our findings raise questions about the commonly agreed conception that electrophysiological attenuations represent a dampened perceived intensity reduction of self-generated stimulations and open the possibility that our perception might be differently prompted by the sensory modality, especially for the somatosensory modality, given its strict link with motor abilities.

## Data Availability

The data that support the findings of this study are available from the corresponding author, GG, taking into account the data protection guidelines. The corresponding scripts for data analysis and for replicating figures are available here: https://github.com/Neurocomputation-and-Neuroimaging-Unit/SensAtt_Aud_Tact_Behav.
